# Social interactions, trust and risky alcohol consumption

**DOI:** 10.1186/s13561-016-0081-y

**Published:** 2016-01-12

**Authors:** Abdu Kedir Seid

**Affiliations:** Centre for Alcohol and Drug Research, Aarhus University, Artillerivej 90, 2. 2300 København S, Denmark

**Keywords:** Risky alcohol consumption, Heavy episodic drinking, Social interaction, Generalised trust

## Abstract

**Background:**

The association of social capital and alcohol consumption is one of the most robust empirical findings in health economics of the past decade. However, the direction of the relationship between the two is heavily dependent on which dimension of social capital is studied and which alcohol measure is used. In this paper, we examine the effect of social interactions and generalised trust on drinking in the general Danish population survey.

**Methods:**

Participants (*n* = 2569) were recruited as part of a larger study. The double-hurdle model for the volume of alcohol consumption and the multivariate logistic model for heavy episodic drinking were estimated.

**Results:**

We found evidence that social networking with male friends, membership in voluntary organisations, and generalised trust were significantly associated with the mean volume of alcohol consumption and heavy drinking. We also observed that social support at the community level had a buffering effect against heavy episodic drinking.

**Conclusions:**

The findings support previous findings in which social interactions and generalised trust were found to predict individuals’ volume of drinking and heavy episodic drinking. However, the results varied across the indicators.

## Background

During the past three decades or so, the concept of social capital has received much attention by practitioners and academicians from different disciplines. The work of Coleman [8] in sociology, Putnam et al. [32] in political science, and Kawachi et al. [24] in public health has contributed to developing a terminology and theoretical framework for the concept. According to Putnam et al. [32], social capital is defined as those features of social organisation such as trust, norms, and networks that facilitate coordinated actions to improve societal efficiency. In his definition, and in many others, the following components are included: social networks, social norms, support, and trust.

There are several mechanisms through which social capital indicators are related to individual health. Social interaction increases the utility of its performers, and it also improves allocation of resources by improving information sharing, coordination of activities, and collective decision making [4]. Bolin and his colleagues also pointed out that an individual with a large social network is more likely to be monitored and controlled relative to individuals with a weak or no social network. In this context, the network regulates and exerts social control over risky health behaviours such as smoking, drinking alcohol, and drug use. Furthermore, the authors argued that individuals who have strong social networks are less likely to feel stressed and engage in health damaging behaviours while individuals who are socially isolated are more likely to engage in risky health behaviours.

Social participation is viewed as strengthening self-esteem and coping strategies as well as facilitating a greater degree of empowerment and accountability [30]. Such enhancements have been shown to be positively associated with better mental health [22]. Similarly, support from friends, family, and health professionals improves health by encouraging healthy behaviours and discouraging health damaging behaviours [9]. Furthermore, individuals with low levels of trust, (also an indicator of social capital) are more likely to be involved in risky health behaviours because they may be more sceptical of taking health-related advice from friends, physicians, and public institutions [27]. In sum, social capital affects individual health by promoting more rapid diffusion of health information, increasing the likelihood that healthy norms of behaviour are adopted, and exerting social control over deviant health-related behaviour [23].

There has recently been a growing interest in the various indicators of social capital as the major correlates of drinking behaviour. Despite mixed results, previous studies documented a significant association between social capital indicators and alcohol use. Some of the empirical evidence suggested that a high level of social capital is protective against engaging in risky health behaviours. For instance, Weitzman and Kawachi [34] found that high social capital is associated with a lower risk of binge drinking among college students in the United States. In contrast, Lundborg [27] found no evidence that social capital (i.e., trust and social participation) is related to binge drinking among Swedish adolescents. However, he did find that social capital is negatively associated with the probability of smoking and illicit drug use. A recent study by Åslund and Nilsson [2] that used Swedish data demonstrated that low neighbourhood social capital is associated with increased alcohol consumption, smoking, and illicit drug use among students.

Taken together, the link between social capital indicators and alcohol use has been demonstrated by the abovementioned studies. However, there are some important shortcomings in the previous literature. Firstly, the majority of the studies relied heavily on dichotomous outcomes of alcohol consumption measures, which might result in a loss of information and loss of statistical power in the results. A few studies even employed least square methods, and in the presence of zero alcohol consumption, this approach generally leads to inconsistent parameter estimates. In the present study, this issue was addressed by using a double-hurdle model that is appealing to investigate participation decision (drinking or not) and the amount or consumption decision (volume of drinking when it is positive). Thus, the model enabled us to not only assess how social capital indicators predict the probability of drinking but also the intensity of alcohol consumption.

Secondly, many studies examining the effect of social capital on alcohol measures used observational data sets. Since social capital indicators are also likely to be influenced by alcohol use, the relationship between the two may be simultaneously determined, in which case the results will be biased and inconsistent. Additionally, Kim et al. [25] pointed out that social capital does not randomly vary among individuals, and the failure to account for unobserved individual characteristics correlated with social capital leads to a spurious statistical relationship. There are, however, some exceptions that addressed this issue through the instrumental variable approach [14, 17, 25]. Furthermore, some studies examined the relationship between social capital and the outcome of interest at the neighbourhood level to mitigate the problem. In this regard, d’Hombres et al. [11] and d’Hombres et al. [12] have instrumented trust, membership, and social isolation by calculating the community averages of the three social capital indicators for each individual as the mean of all other individuals living in the same community. Similarly, we addressed the simultaneity problem through measuring social capital indicators by taking the average prevalence of the indicators across the individual’s postal code area after the person’s contribution to the mean was subtracted. In this regard, the present study sought to provide a new perspective on the relationship between social capital indicators and alcohol consumption using an approach commonly used to study peer effects [28].

## Methods

### Data and measures

Data for the present study came from the 2011 Danish alcohol and drug survey that was carried out by Statistics Denmark for the Centre for Alcohol and Drug Research of Aarhus University. A representative sample of the Danish general population aged 16 to 79 years old was randomly chosen using the central person registry (CPR) as a sampling frame. Among this, we used 2569 individuals, who were randomly selected and additionally interviewed on dimensions of social capital.

We used *volume of alcohol consumption* and *heavy episodic drinking* as outcome variables. Respondents were asked how many glasses or units of the various types of alcoholic beverages (beer, wine, spirits, and cider) they drank in the last 30 days. These beverage-specific quantity-frequency measures were then summed up to create a measure of total alcohol consumption in grams of pure alcohol on an average per day. Heavy episodic drinking was defined as drinking five or more units on one occasion in the previous 12 months. It was a dummy variable taking 1 if the individual engaged in heavy episodic drinking and 0 otherwise.

*Social interaction* among friends and colleagues was derived from the following questionnaire item: ‘How often have you had interaction (including e-mails, letters, and text messages) with colleagues, female friends, and male friends in the last 30 days?’. Response categories ranged from ‘daily (5)’ to ‘have no relative/colleague (0)’. *Social support* was constructed based on the following questionnaire item: ‘How easy or difficult is it to get help from friends if you need it?’. Response categories ranged from ‘4’ representing ‘very easy’ to ‘1’ representing ‘very difficult’. As for indicators of *social participation,* a dummy variable was created for the question which reads: ‘Are you a member of an organisation, club, or other organisations?’. Finally, *generalised trust* was measured with the following item: ‘To what extent do you trust most people?’ The response category ranged from ‘to a high extent’ (4) to ‘never’ (1).

The model also included the following variables: Age (15–29, 30–45, 46–64, and 65 years and older); gender; personal annual income (categorised into five groups: the lower category was 100,000 Danish kroner (kr.) or less (ca. US$ 16,987), and the upper category was 400.000 kr. (Ca. US$ 67,949) and more; education (basic schooling, upper secondary and vocational, and post-secondary); employment status (employed and self-employed, student, pensioner, unemployed, and other including home maker); civil status (married/living in a relationship, divorced/separated/widowed, single); and a dummy for living with children under 18 years old.

### Econometric analysis

#### Volume of alcohol consumption

In our sample, about 7 % of respondents were abstainers, and in the presence of censoring (i.e., zero alcohol consumption), least square estimates will be inconsistent. Among a number of available estimation procedures, the double-hurdle model proposed by Cragg [10] can be used to account for this non-drinking problem. Thus, this method was used to predict social capital indicators on the volume of drinking. In the present case, individuals are assumed to make their consumption decisions in two steps. First, the individual decides whether to drink or not. This is referred to as the ‘participation decision’. If the participation decision is positive, the individual then decides upon an optimal consumption amount given his or her condition at the time, and this is called the ‘consumption decision’ [16]. The double-hurdle model has been previously applied in the area of alcohol and tobacco consumption [1, 13, 33, 35].

Following Aristei et al. [1], we used the following specification to estimate the likelihood and intensity of alcohol consumption:

(i) Observed equation: *y* = *d* × *y* * *
(ii) Participation equation: $$ \begin{array}{l}p={\varphi}^{\hbox{'}}w+u\kern0.5em ,\kern1em u\sim N\left(0,1\right)\\ {}d=\left\{\begin{array}{c}\hfill 1\  if\kern0.75em p>0\hfill \\ {}\hfill \kern0.75em 0\  otherwise\hfill \end{array}\right.\end{array} $$
(iii) Consumption equation: $$ \begin{array}{l}y*={\beta}^{\hbox{'}}x+v,\ v \sim N\left(0,{\sigma}^2\right)\\ {}\kern0.5em y**=\left\{\begin{array}{c}\hfill y* if\ y* > 0\hfill \\ {}\hfill 0\  otherwise\hfill \end{array}\right.\end{array} $$

Where: *w* and *x* are factors including the social capital indicators that are likely to influence participation and consumption decisions, respectively. The error terms in the two decision equations are given by *u* and, respectively. A positive level of alcohol consumption *(y* = *y*∗*)* is only observed if the individual participates in drinking *(d* = 1*)* and actually consumes alcoholic beverages *(y*∗ *>* 0*)*. In this model, we assumed that the decision to drink and the amount to drinking (consumption) are independent.

#### Heavy episodic drinking

As mentioned earlier, heavy episodic drinking was a dichotomous variable taking the value 1 if the individual participated in the outcome of interest and 0 otherwise. In this regard, multivariate logistic regression was used to predict social capital indicators on heavy episodic drinking. In the spirit of Lundborg [28], for an individual *i* in postal code area *z*, the average social capital indicators excluding own contribution was constructed as follows:

$$ Social\  capita{l}_{iz}=\frac{1}{N-1}{\displaystyle \sum_{\begin{array}{c}\hfill j=1\hfill \\ {}\hfill j\ne i\hfill \end{array}}^N}{y}_j $$

Where: *N* is the number of respondents in Z postal code area and *y*_*j*_ is a Likert scale or binary indicator (for membership in organisation) value representing responses of an individual *j*. For an individual *i*, the heavy episodic drinking decision can be defined as:

$$ Heav{y}_i^{*}={\beta}_0+{\beta}_1{X}_{1i}+{S}_i+{\varepsilon}_i $$

The latent variable $Heav{y}_i^{*}$ takes the value 1 if $Heav{y}_i^{*}>0$ and zero otherwise. *X*_1*i*_ represents the vector of factors that is likely to influence heavy episodic drinking, *S*_*i*_ represents the average social capital indicators excluding the individual contribution, *β*_*j*_ is the associated vector of coefficients, and *ε*_*i*_ is the random error term.

With respect to the validity of the constructed models, the pair-wise correlation coefficient test and the variance inflation factor (VIF) test could not confirm multicollinearity in any of our tested equations (see Appendix 1 and 2). The STATA 13.1 software package was used for all of the analyses.

## Results

Table 1 displays the descriptive statistics of variables used in the analyses. The mean volume of alcohol consumption per drinking day was about 12 g of pure alcohol, while less than a fifth of individuals reported participating in heavy episodic drinking in the previous 12 months. More individuals had social interaction with colleagues followed by interactions with female and male friends. Furthermore, individuals on average reported high social support whereas more than half of the respondents were members in an organisation. Looking at the socioeconomic and demographic variables, most respondents identified themselves as middle aged, better educated, employed, married, or living with a partner.

**Table 1:** Frequency distribution of study variables

Variable	Mean	Standard deviation	*p*-values
Alcohol consumption (mean volume; grams of pure alcohol per day)	12.28	19.05	
Heavy episodic drinking (%)	0.70	0.46	
Social interaction with			
Colleague	3.06	2.09	0.22***
Female friend	2.98	1.51	0.02
Male friend	2.74	1.53	0.24***
Social support	3.43	0.65	0.06**
General trust	3.19	0.71	0.07***
Membership in organisation	0.57	0.49	0.08***
Age group			
15–29	18.3 %		
30–45	26.8 %		
46–64	36.9 %		
65+	18.1 %		
Income			
≤100,000	15.1 %		
100,000–199,999	21.6 %		
200,000–299,999	21.3 %		
300,000–399,999	20.4 %		
≥400,000	21.8 %		
Education			
Low	22.5 %		
Middle	37.3 %		
High	40.2 %		
Employment			
Employed	55.7 %		
Student	10.1 %		
Unemployed	2.3 %		
Pensioner	24.2 %		
Other	7.7 %		
Civil status			
Married/lived with partner	73.4 %		
Widowed/divorced/separated	10.5 %		
Single	16.0 %		
Lived with under 18 child	0.32	0.47	0.05*

*Note*: Mean value and standard deviations are reported for drinking, activity in organisation, and family status variables. The p-values are for spearman correlation performed between heavy episodic drinking and the respective variables

*p < 0.05;**p < 0.01;***p < 0.001

### Volume of alcohol consumption

As noted earlier, the double-hurdle model uses two equations: participation and consumption equations. In the former, all non-participants have zero alcohol consumption, whereas participants have a positive volume of alcohol consumption in the latter. The two equations are estimated simultaneously using the user written STATA command by Burke [5]. The same covariates were controlled in the two equations despite the fact that the model allows us to use different covariates in both equations. To understand the overall effect of the covariates in the first and second hurdles, the average partial effect (APE) of the estimated covariates with bootstrap standard errors are calculated [5]. Table 2 presents the results of the hurdle model, and the reported quantities include the APE and its 95 % confidence intervals. In the first column, the results of the first hurdle, i.e., the participation equation, are presented, whereas the second column reports the intensity of alcohol consumption. As shown in the table, most of the estimated coefficients of the covariates in the participation equation have the same sign as those in the quantity equation except the estimated coefficients of social support and age variables. Social networking with male friends was positively associated with the number of drinks consumed during the past 12 months but not the likelihood of drinking. Membership in an organisation and generalised trust appeared to be positively related to both the likelihood of drinking and the number of drinks consumed. The results on socioeconomic and demographic variables show that the probability of drinking and the number of drinks consumed increased among individuals with high education and income in comparison to individuals in the lower groups. It is also observed that individuals who identified themselves as pensioners tended to consume less compared to those who were employed. Furthermore, individuals who reported having children had significantly lower probability of participating in drinking, but if they drink, they tend to drink less in comparison to respondents without children.

**Table 2:** Average Partial Effects of the likelihood and intensity of alcohol consumption

Variables	First hurdle (participation)	Second hurdle (alcohol consumed)
Social networking with		
Colleague	0.00(0.00)	0.00(0.01)
Female friend	0.00(0.00)	0.00(0.00)
Male friend	0.00(0.00)	0.03(0.01)**
Social support	−0.00(0.01)	0.02(0.02)
Membership in organisation	0.02(0.01)*	0.05(0.01)**
General trust	0.02(0.01)****	0.06(0.01)***
Gender (female = 1)	−0.03(0.01)*	−0.25(0.02)***
Age group (ref. 15–29)		
30–45	0.01(0.02)	−0.09(0.04)*
46–64	0.01(0.02)	−0.02(0.05)
65+	0.03(0.02)	0.09(0.05)
Education (ref. low)		
Middle	0.03(0.01)**	0.06(0.03)*
High	0.04(0.01)**	0.06(0.03)*
Income (ref. ≤100,000)		
100,000–199,999	0.02(0.01)	0.01(0.04)
200,000–299,999	0.03(0.02)	0.08(0.03)*
300,000–399,999	0.08(0.11)**	0.11(0.04)*
≥400,000	0.07(0.02)*	0.13(0.09)*
Employment (ref. employed)		
Student	0.01(0.01)	0.05(0.03)
Unemployed	0.01(0.07)	−0.09(0.06)
Pensioner	−0.03(0.02)	−0.10(0.05)*
Other	−0.02(0.02)	−0.02(0.02)
Civil status (ref. married or live with partner)		
Widowed/divorced/separated	−0.04(0.02)*	−0.05(0.04)
Single	0.01(0.02)	0.04(0.03)
Lived with under 18 children	−0.04(0.01)**	−0.15(0.03)***
*N*	2213	
*Log likelihood at convergence*	-1442.5337	

*Note*: Bootstrap standard errors are in parentheses. Number of observation and the log likelihood values were obtained from the preliminary estimation

Sig. **** p < 0.10;*p < 0.05;**p < 0.01;***p < 0.001

In sum, the results suggest that membership in an organisation and generalised trust were positively associated with both the likelihood of alcohol use and the number of drinks consumed in the previous year. Furthermore, social networking with male friends was positively related to the number of drinks consumed.

### Heavy episodic drinking

The results of the multivariate logistic regression examining heavy episodic drinking as an outcome are reported in Table 3. The results showed that, except for social support, the estimated coefficient for the social interactions and generalised trust variables at the community level have little effect on engaging in heavy episodic drinking. In the results, the social support variable appeared to be negative and statistically significant. This implies that a 10 % point increase in average social support in the community was likely to decrease the probability of an individual’s heavy episodic drinking by 0.6 % points.

**Table 3:** Results of multivariate logistic regression

	Average marginal effects (S.E.)
Social networking with	
Colleague	−0.01(0.01)
Female friend	−0.01(0.01)
Male friend	0.01(0.01)
Social support	−0.06(0.03)*
Membership in organisation	0.05(0.04)
General trust	−0.01(0.03)
Gender (female = 1)	−0.19(0.02)***
Age group (ref. 15–29)	
30–45	−0.15(0.04)**
46–64	−0.21(0.04)***
65+	−0.21(0.05)***
Education (ref. low)	
Middle	0.03(0.02)
High	0.05(0.03)
Income (ref. ≤100,000)	
100,000–199,999	0.04(0.03)
200,000–299,999	0.06(0.04)
300,000–399,999	0.12(0.04)**
≥400,000	0.17(0.04)***
Employment (ref. employed)	
Student	0.00(0.02)
Unemployed	−0.14(0.06)
Pensioner	−0.02(0.03)***
Other	−0.06(0.01)***
Civil status (ref. married or live with partner)	
Widowed/divorced/separated	−0.06(0.03)*
Single	−0.02(0.03)
Lived with under 18 children	−0.05(0.03)*
*Pseudo R2*	0.13
*N*	2253

Sig. *p < 0.05;**p < 0.01;***p < 0.001

As seen in the results, women were less likely to engage in heavy episodic drinking compared to men. The probability of engaging in heavy episodic drinking appeared to decrease among individuals in the older age cohorts in comparison to individuals in the young age cohort. The results for the estimated coefficients of the socioeconomic variables were qualitatively comparable. For instance, the result indicates that income had a significant positive effect on the probability of heavy episodic drinking. We also found that individuals who identified themselves as pensioners and in the ‘other’ employment category were less likely to engage in heavy episodic drinking compared to their counterparts in the employed category. Civil status is also related to heavy episodic drinking: Individuals who identified themselves as widow/divorced/separated were likely to engage in heavy episodic drinking compared to married individuals. With respect to children, individuals who reported having children were less likely to engage in heavy episodic drinking compared to those who did not live with children.

In sum, the results suggest that after controlling for socioeconomic and demographic variables social support at the community level, which is defined as community effects elsewhere [29, 36], was found to be significantly associated with the likelihood of heavy episodic drinking.

## Discussion

The current study examined social interactions and generalised trust as predictors of alcohol use among a representative sample of Danish adults. We used the double-hurdle model to not only explore the probability of drinking but also the intensity of alcohol consumption. Furthermore, to address the simultaneity issue, we used the average value of the social interaction and generalised trust variables by excluding own contribution to predict the likelihood of heavy episodic drinking. Our result demonstrates that social interactions and generalised trust were significantly correlated with alcohol use measures.

With respect to our first outcome measure, i.e., volume of alcohol consumption, we found that social networking with male friends was positively associated with the volume of drinking. Our finding supports the qualitative research by Järvinen [21] who reported that alcohol plays a considerable role in Danish social interactions. Furthermore, our result is consistent with Bloomfield et al. [3] who found that indicators of social interactions are more strongly associated with Danish drinking than classical socio-economic factors. We also found that membership in an organisation positively predicts the likelihood and intensity of drinking. The result implies how alcohol consumption may play a common part of socialisation, and it agrees with studies conducted in other countries that documented positive correlation between social participation and risky drinking [6, 29, 31]. Similarly, our results reveal that generalised trust increased the intensity of alcohol consumption. This is contrary to previous findings by Lundborg [27] who did not observe trust predicting binge drinking among Swedish adolescents and by Lindstrom [26] who found that low levels of generalised trust were associated with high alcohol consumption among male respondents in Sweden. An explanation for this discrepancy may include the fact that the other studies were based on a sample of adolescents or that they used different trust and alcohol measures. Our results for the socioeconomic and demographic variables are in line with Grittner et al. [18] who found that high levels of education and income were associated with higher alcohol consumption in Denmark.

Focussing on heavy episodic drinking as an outcome variable, the same general findings are evident for our examination of social interactions and generalised trust variables in relation to the frequency of engaging in heavy episodic drinking. When analysing the average social capital indicators, i.e., community effects, independently, we found that individuals who reported high social support were less likely to engage in heavy episodic drinking. Thus, our result suggests that adequate support may help as a buffering mechanism against risky health behaviour by reducing stressful actions [7]. It is also evident that support from friends, family, and health professionals can augment physical health by encouraging health-promotion behaviour and discouraging poor health-related behaviours, whereas a lack of positive support can lead to overindulgence in risky behaviours [9]. Our weak results for the effect of the average values of social interaction, membership, and trust variables on heavy episodic drinking partially agree with previous findings from other egalitarian societies, i.e., Sweden. Åslund and Nilsson [2] argued that having strong social autonomy and being less dependent on other individuals such as neighbours and relatives in Sweden could explain the weak correlation between social capital at the contextual level and alcohol consumption.

Furthermore, consistent with earlier research in Denmark and elsewhere, age and being female were observed to be inversely associated with heavy episodic drinking [3]. Our finding for the income variable agrees with previous studies conducted by Hansen et al. [20] who found that household income was associated with heavy episodic drinking in Denmark. Contrary to this, using the 2003 Danish survey data, Bloomfield et al. [3] reported that there was no significant association between monthly personal income and heavy episodic drinking. Although differences in income measures and sample setting in the studies may explain the mixed results, further investigation is warranted to elucidate the influence of income on heavy episodic drinking. The protective effect of living with children against heavy episodic drinking found in this study is consistent with Hansen et al. [20] who found a higher prevalence of heavy episodic drinking among persons without children in Denmark.

It was observed that the results for the social interactions and generalised trust variables were somewhat consistent across the outcome variables. However, except for social support, the magnitude and significance of the results tended to attenuate when we control for average social capital indicators in relation to heavy episodic drinking.

## Conclusions

In conclusion, this study examined the role of social interactions and generalised trust in alcohol consumption while controlling for other important socioeconomic and demographic variables. The effect of social capital has been conceptualised to be inversely associated with alcohol use. However, our results have provided no conclusive support to the argument that individuals who reported higher levels of social capital are less likely to be involved in drinking than their counterparts with less social capital. In this regard, while our result for social support and membership in an organisation supports the argument put forward in the introduction section of the study, the findings from the other aspects of the social capital indicators are observed as working in the opposite direction. Therefore, further studies should be conducted to support these findings and extend the research.

There are a number of limitations that must be acknowledged. Firstly, the study was cross-sectional, which limits interpretations to associations rather than causality. Furthermore, a reverse causation may arise because a person is likely to drink with the aim of socialisation, and, thus, tends to have more social networking and drink more. Additionally, as social networking may influence heavy drinking, the reverse could be true as heavy drinkers may be expected to be isolated and consequently lose friends. However, we addressed this issue partially by considering the average values of the variable of interest at the community level. Secondly, because of the use of self-report data in our analyses, we are aware of the risk of recall bias. Thirdly, despite controlling for a number of individual socio-economic and demographic covariates, the possibility of respondents choosing friends who are similar to them in terms of drinking was not accounted for; that is, we could not control for friends’ drinking behaviour. Furthermore, gender difference may also be important when the influence of friends is considered. Male friends may have a different influence on men than women and female friends may also have a different influence on men than women. For instance, women may fear the embarrassment of being seen drunk by women, but men may take pride in showing other men how much they can drink. Fourthly, we analysed social networking at the friends’ level, but we were unable to take into account the drinking behaviours across contexts. For instance, respondents may drink more at bars than at home or vice-versa [15]. In one study in Denmark, for example, heavy drinkers more often reported drinking at home with family/friends than in licenced areas [19].

In sum, this study examined how social interactions and generalised trust could influence alcohol consumption, controlling for other important socioeconomic and demographic variables. Our main finding is that social interactions and generalised trust were found to predict individuals’ volume of drinking and heavy episodic drinking. However, the results varied across the indicators. It is also worth mentioning that our estimated results are not interpreted as causal relationships, and it is therefore of interest to investigate the causal relationships in future studies.

## Appendix

### Appendix 1

**Table 4:** Variance inflation factor values for study variables

Variable	VIF
Social interaction with	
Colleague	2.33
Female friend	1.34
Male friend	1.38
Social support	1.10
General trust	1.13
Membership in organisation	1.10
Age group (15–29 = REF)	
30–45	3.88
46–64	4.31
65+	4.62
Income (≤100,000 = REF)	
100,000–199,999	2.80
200,000–299,999	3.25
300,000–399,999	3.67
≥ 400,000	4.13
Education (Low = REF)	
Middle	1.87
High	2.20
Employment (Employed = REF)	
Student	2.32
Unemployed	1.15
Pensioner	4.05
Other	1.15
Civil status (Married/lived with partner = REF)	
Widowed/divorced/separated	1.10
Single	1.57
Lived with under 18 children	1.70
Mean VIF	2.37

### Appendix 2

**Table 5:** Pairwise correlation among study variables

Variable	(1)	(2)	(3)	(4)	(5)	(6)	(7)	(8)	(9)	(10)	(11)	(12)	(13)
Alcohol consumption (1)	1.000												
Interaction with colleague (2)	0.072	1.000											
Interaction with female friend (3)	−0.029	0.129	1.000										
Interaction with male friend (4)	0.199	0.213	0.384	1.000									
Social support (5)	0.077	0.075	0.177	0.163	1.000								
General trust (6)	0.099	0.097	0.104	0.042	0.190	1.000							
Membership in organisation (7)	0.108	0.031	0.045	0.046	0.066	0.146	1.000						
Age group (8)	−0.008	−0.338	−0.289	−0.333	−0.037	0.058	0.136	1.000					
Income (9)	0.093	0.459	−0.139	−0.023	0.009	0.173	0.137	0.122	1.000				
Education (10)	0.028	0.216	0.022	−0.048	0.008	0.213	0.186	0.045	0.386	1.000			
Employment (11)	−0.066	−0.611	−0.068	−0.128	−0.045	−0.078	−0.011	0.347	−0.430	−0.157	1.000		
Civil status (12)	0.061	−0.067	0.247	0.248	−0.016	−0.063	−0.018	−0.269	−0.283	−0.157	0.036	1.000	
Lived with under 18 children (13)	−0.154	0.284	0.029	−0.015	−0.020	0.084	0.014	0.245	0.294	0.203	−0.303	−0.245	1.000
